# Proactive and reactive inhibitory control in rats

**DOI:** 10.3389/fnins.2014.00104

**Published:** 2014-05-08

**Authors:** Jeffrey D. Mayse, Geoffrey M. Nelson, Pul Park, Michela Gallagher, Shih-Chieh Lin

**Affiliations:** ^1^Department of Psychological and Brain Sciences, Johns Hopkins UniversityBaltimore, MD, USA; ^2^Neural Circuits and Cognition Unit, Laboratory of Behavioral Neuroscience, National Institute on Aging, National Institutes of HealthBaltimore, MD, USA; ^3^Neurocognitive Aging Section, Laboratory of Behavioral Neuroscience, National Institute on Aging, National Institutes of HealthBaltimore, MD, USA

**Keywords:** stop signal, stop signal reaction time, reactive, proactive, inhibitory control, impulsivity, executive function, decision making

## Abstract

Inhibiting actions inappropriate for the behavioral context, or inhibitory control, is essential for survival and involves both reactively stopping the current prepared action and proactively adjusting behavioral tendencies to increase future performance. A powerful paradigm widely used in basic and clinical research to study inhibitory control is the stop signal task (SST). Recent years have seen a surging interest in translating the SST to rodents to study the neural mechanisms underlying inhibitory control. However, significant differences in task designs and behavioral strategies between rodent and primate studies have made it difficult to directly compare the two literatures. In this study, we developed a rodent-appropriate SST and characterized both reactive and proactive control in rats. For reactive inhibitory control, we found that, unlike in primates, incorrect stop trials in rodents result from two independent types of errors: an initial failure-to-stop error or, after successful stopping, a subsequent failure-to-wait error. Conflating failure-to-stop and failure-to-wait errors systematically overestimates the covert latency of reactive inhibition, the stop signal reaction time (SSRT). To correctly estimate SSRT, we developed and validated a new method that provides an unbiased SSRT estimate independent of the ability to wait. For proactive inhibitory control, we found that rodents adjust both their reaction time and the ability to stop following failure-to-wait errors and successful stop trials, but not after failure-to-stop errors. Together, these results establish a valid rodent model that utilizes proactive and reactive inhibitory control strategies similar to primates, and highlight the importance of dissociating initial stopping from subsequent waiting in studying mechanisms of inhibitory control using rodents.

## Introduction

Inhibitory control, or the ability to inhibit actions inappropriate for the context, is essential for meeting the shifting demands of complex environments (Logan et al., 1997). Successful inhibitory control can be achieved through both proactive and reactive control strategies, respectively involving preparation to stop prior to stimulus onset and stimulus-driven processing at stimulus onset (Li et al., 2006a; Aron, 2011). One important paradigm to study inhibitory control is the stop signal task (SST). In the SST, subjects need to rapidly cancel a prepotent behavioral response when the go signal is occasionally followed by a stop signal. The SST is uniquely powerful in that it allows for the quantitative estimation of the latency of reactive inhibition, the stop signal reaction time (SSRT) (Logan and Cowan, 1984; Logan et al., 1984). Subjects also employ proactive inhibition in the SST to adjust response speed following errors or stop trials (Emeric et al., 2007; Verbruggen and Logan, 2009; Bissett and Logan, 2012). Understanding the neural mechanisms of inhibitory control is critical because elevated SSRT is a widely observed feature of cognitive impairment across many neuropsychiatric disorders, including Parkinson's disease (Gauggel et al., 2004; Mirabella et al., 2011), attention-deficit hyperactivity disorder (McAlonan et al., 2009), and normal cognitive aging (Andrés et al., 2008; Coxon et al., 2012; Hu et al., 2013).

Recent years have seen a surging interest in rodent versions of the SST (Eagle and Robbins, 2003a; Bryden et al., 2012; Leventhal et al., 2012) to leverage the advantages of rodent models, such as lesion, pharmacology and recording. However, important differences exist between current rodent and primate SSTs and pose a major challenge in comparing the two literatures. For example, while primates typically need to cancel the initiation of an action, rodents are commonly required to inhibit an ongoing movement (Eagle and Robbins, 2003a; Bari et al., 2011; Bryden et al., 2012; Beuk et al., 2014). Furthermore, while primate SSTs typically use multiple stop signal delays (SSDs), the intervals between go and stop signal onset, many rodent tasks employ only a single SSD (Leventhal et al., 2012; Schmidt et al., 2013), encouraging rats to adopt an alternative timing strategy in anticipation of the highly predictable stop signal. Finally, the implications of different behavioral strategies between rodents and primates, such as differing baseline response biases, on estimating SSRT have not been systematically investigated (Robinson et al., 2009). Reconciling these differences is an essential step toward establishing the rat as a valid model to study inhibitory control.

The goal of this current study was to examine whether rats exhibit proactive and reactive inhibitory control as commonly described in primates in a novel rodent-appropriate SST. Specifically, we investigated how rats cancel the initiation of a rapid nosepoke port exit response while incorporating multiple SSDs within each session. We found that, in addition to errors of stopping, rats often commit independent errors of waiting. The conflation of these two types of errors using current estimation methods systematically overestimates SSRT. This led us to develop and validate a novel method to estimate SSRT independent of the ability to wait. We also investigated how recent trial history leads to proactive adjustments of subsequent reaction time and the ability to stop.

## Methods

### Subjects

Ten male Long-Evans rats (Charles River, NC), weighing 250–350 g at the start of the experiment, were trained in this study. Animals were housed individually in a temperature- and humidity-controlled vivarium on a 12L:12D cycle (lights on at 0700). Animals were provided *ad libitum* access to water and food restricted to 85% of their free-feeding weight to motivate behavioral training. Subjects were trained in one daily 90 min session. Six of these animals underwent stereotaxic surgery for implantation of chronic recording electrodes for a separate study. All experimental procedures were conducted in accordance with the National Institutes of Health (NIH) Guide for the Care and Use of Laboratory Animals and approved by the National Institute on Aging Animal Care and Use Committee.

### Apparatus

Plexiglas operant chambers (11″L × 8 ¼″W × 13″H), custom-built by Med Associates Inc. (St. Albans, VT) were contained in sound-attenuating cubicles (ENV-018MD) each with an exhaust fan that helped mask external noise. Each chamber was equipped with one photo-beam lick-o-meter reward port (CT-ENV-251L-P) located in the center of the front panel, with its sipper tube 7.5 cm above the grid floor. Two infrared (IR) sensors were positioned to detect reward port entry and sipper tube licking, respectively. Diluted liquid sweetened condensed milk (2:1 water:milk) was used as reward and delivered through a custom-built multi-barrel sipper tube. The delivery system was controlled by pressurized air and each solenoid opening (10 ms) was calibrated to deliver a 10 μl drop of fluid. The reward port was flanked by two nosepoke ports (ENV-114M), located 6.6 cm to each side and 5.9 cm above the grid floor. Each nosepoke port was equipped with one IR sensor to detect port entry. Only the right nosepoke port was used in behavioral training as the fixation port, while the left nosepoke port was inactive but remained available for exploration.

Each chamber was equipped with two ceiling-mounted speakers (ENV-224BM) to deliver auditory stimuli, and two stimulus lights (ENV-221) positioned above the reward port in the front panel. Behavior training protocols were controlled by Med-PC software (Version IV), which stored all event timestamps at 2 ms resolution. All behavioral sessions were recorded via overhead video cameras and data were stored offline for later analysis.

### Behavioral shaping

#### Fast response to the go signal

Rats were initially trained to respond to a 6 kHz tone (2 s, 80 dB) in the operant chamber to receive 3 drops of reward (30 μl) in the reward port, delivered starting at the 3rd lick. Only trials with the three licks within a 3 s reward window were considered successful and rewarded. Subsequently, rats were shaped to nosepoke in the fixation port and maintain fixation until tone presentation. Early fixation port exit before tone onset resulted in no reward delivery. The delay between fixation port entry and tone onset, or foreperiod, was adaptively increased until rats could reliably maintain fixation for 800 ms in anticipation of the tone. After that point, four different foreperiods (350, 500, 650, and 800 ms) were used, varied pseudo-randomly across trials, to minimize temporal expectation of stimulus onset. The inter-trial interval (ITI) was 1–3 s, and not signaled to the rat. Premature fixation port entry and premature licking during ITI both reset the ITI timer. A cutoff of 500 ms reaction time (RT), the latency between tone onset and fixation port exit, was also imposed such that RTs longer than the 500 ms were not rewarded. Animals were held at this stage for 10–14 sessions to encourage fast responding to the tone, until 90% of RTs were faster than 500 ms.

#### Withhold response to the light signal

After rats were trained to respond as fast as possible to the 6 kHz go signal, they were trained to associate a light signal with reward if they responded after the light offset but not before. The overall organization of the task was the same as the previous shaping phase, except that the 6 kHz tone was replaced by illumination of a white central panel light in each trial, which will later serve as the stop signal in the SST. Fixation port exit responses during light illumination led to forfeiture of reward, and only responses after light offset led to 3 drops of reward (30 μl). The duration of light illumination was initially set at 350 ms, such that some fixation port exit responses were slow enough to be rewarded. To provide an explicit feedback to animals that they had waited long enough in the fixation port and that reward was available, waiting for the duration of the light was coupled with an audible solenoid click similar to the click associated with reward delivery. The light duration was then adaptively increased until animals could reliably wait for 500 ms light illumination (10–14 sessions). After that, rats received several refresher tone-alone sessions before transitioning to the SST.

### Stop signal task (SST)

**Figure 1:**
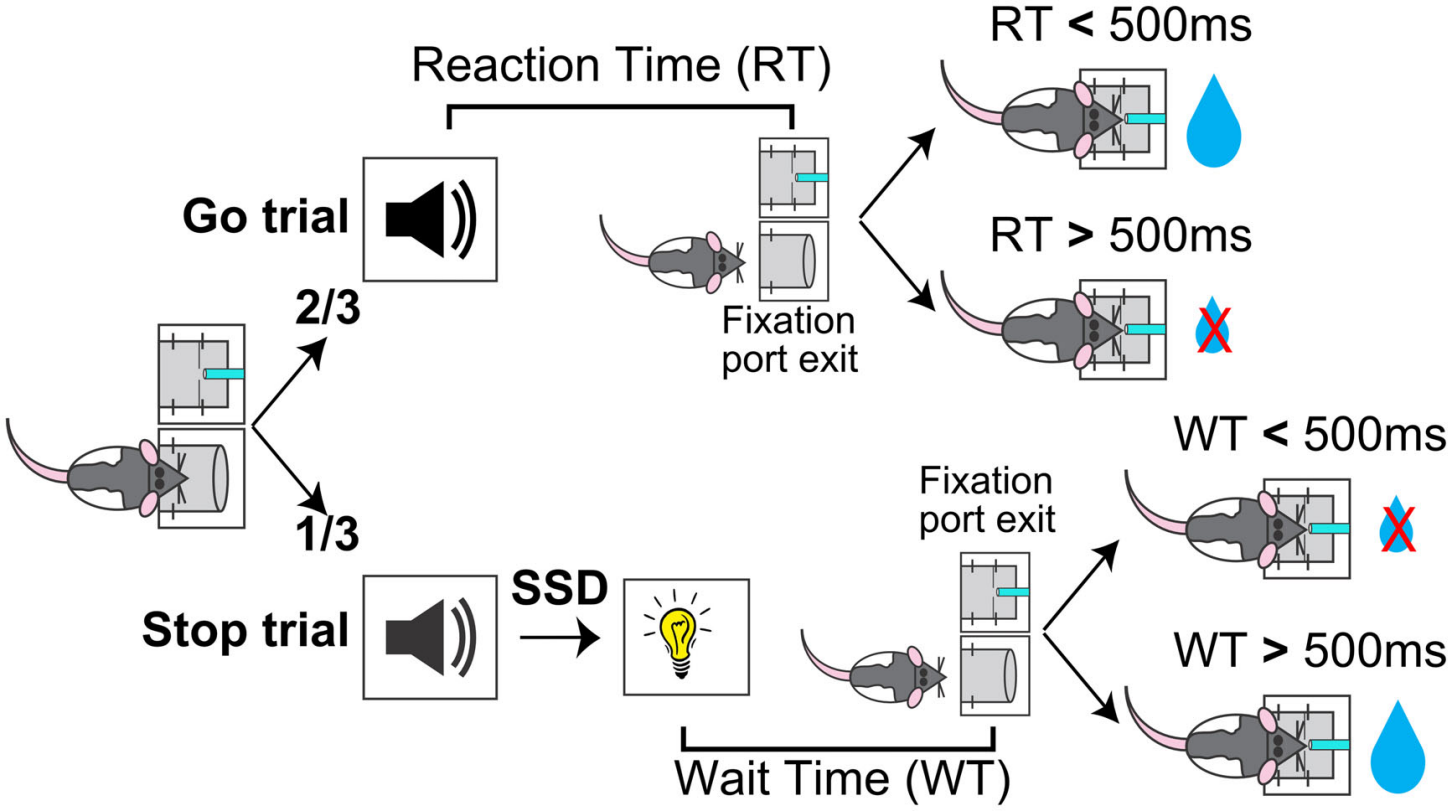
**Schematic of the rodent Stop Signal Task (SST)**. Rats began each trial by inserting their nose into a fixation port. After maintaining fixation for a variable foreperiod, a tone was presented. Two-thirds of trials were Go trials, in which the tone was presented alone and the trial was rewarded if reaction time (RT, latency between tone onset and fixation port exit) was faster than 500 ms. One-third of trials were Stop trials, in which the tone was followed by a Stop light and the trial was rewarded if wait time (WT, latency between light onset and fixation port exit) was longer than 500 ms. The delay between the Go tone and Stop light, the Stop Signal Delay (SSD), varied between trials.

The general organization of the SST, including ITI, nosepoke port fixation, foreperiod and reward delivery, was the same as the two behavioral shaping phases. In the SST, the 6 kHz go signal was presented on all trials, and on 1/3 of the trials the go signal was followed by the light stop signal after a variable stop signal delay (SSD) (Figure 1). In the tone-alone trials (2/3), or Go trials, animals were required to make fast go responses (RT < 500 ms) to receive reward, the same contingency as in the shaping phase. In the Stop trials (1/3), reward contingency was dictated by the stop light as in the shaping phase, such that reward was available only if wait time (WT), the latency between stop signal onset to fixation port exit, was longer than the 500 ms hold duration. The same amount of reward (30 μl) was delivered in both fast Go trials and successful Stop trials. Five SSDs were determined before the start of each session based on performance in the previous session, and the SSD was chosen pseudorandomly from these five SSDs on each trial. Every session included a 0 ms SSD such that the tone and light were presented simultaneously. The remaining four SSDs were evenly spaced in 40 ms steps and adjusted by experimenters between sessions to ensure approximately 50% failed stop trials.

In Stop trials where rats already made the fixation port exit response before the onset of stop signal (RT < SSD), we chose to omit the stop signal and rewarded the animal for the go response in order to encourage fast responses to the go signal. While animals subjectively perceived these “converted” Stop trials as Go trials, these trials were treated as failure-to-stop errors in our analysis to ensure that the stochastic go process was sampled equally in both Go and Stop trials. This is important because these converted trials tended to have very fast RTs: classifying these trials as Go trials would result in a disproportionately fast Go trial RT distribution and slow Stop trial RT distribution from essentially transferring the fastest Stop trials to Go trials. The effect of such a misclassification would be to bias the Go RT distribution leftward (i.e., faster) and under-estimate the percentage of failure-to-stop errors, resulting in the under-estimation of SSRT (i.e., faster SSRT). Note that, because converted trial RTs were faster than the SSD, classifying these trials as failure-to-stop errors resulted in negative values when aligned to the Stop signal (Figure 4). These converted Stop trials were not included when determining the percentage of attempted reward collection in failure-to-stop errors (Figure 3B) and in the analysis of proactive inhibitory control (Figure 7), where only trials with stop signal presentation were included. Previous studies in rodents have treated these trials similarly by omitting stop signal presentation and rewarding fast go responses (Eagle and Robbins, 2003a; Beuk et al., 2014), with the exception of one study which did not address these trials directly (Schmidt et al., 2013).

### Data analysis

Data analysis included only sessions with (1) 50 or more Stop trials; (2) greater than 25% failed Stop trials; and (3) fewer than 25% Go trials with RTs exceeding the 500 ms cutoff (*n* = 257/507 sessions, 49% sessions excluded). Of all excluded sessions, 48% occurred in the initial 1/3 of training days, while only 10% were from the last 1/3 of training days. Data were analyzed using custom-written MATLAB scripts and reported as mean ± s.e.m. All comparisons were conducted on a per subject basis (*n* = 10) except for the correlation analysis (*n* = 257 sessions). *Post-hoc* comparison of means was conducted using paired *t*-tests with Bonferroni's correction for multiple comparisons (α/*n*, where α = 0.05 and *n* is the number of pairwise comparisons conducted). Analysis of error rates and RTs was conducted using repeated measures ANOVA (Figure 3). Correlations (Figures 5C,D, 6B,C) were conducted using Pearson's r. SSRT estimates from simulated data (100 iterations, Figure 5B) were randomly grouped into 10 10-iteration blocks and compared using repeated measures ANOVA (Method × Percent failure-to-wait Error Included) and planned *post-hoc t*-tests corrected for multiple comparisons. Empirical SSRT data (Figure 6A) were compared using paired *t*-tests corrected for multiple comparisons. Comparisons of proactive RT adjustments (Figures 7A,B) were conducted by paired or independent samples *t*-tests as appropriate. In addition, median-normalized RT on G1 and G2 trials (Figure 7B) were compared using One-Way ANOVA and planned *post-hoc t*-tests with Bonferroni's correction for multiple comparisons.

### A new method of estimating SSRT independent of failure-to-wait errors

Our new method aims to estimate SSRT by directly comparing RT distributions in Stop trials and Go trials in order to determine the time point at which the stop signal begins to slow down RTs relative to Go trial RTs (Figure 4). The idea that SSRT estimates the relatively fixed latency for the brain to process the stop signal and cancel the planned go response (Logan et al., 1984) predicts that RTs faster than this fixed latency should be statistically indistinguishable on Stop and Go trials, while RTs longer than this fixed latency should be much slower on Stop than Go trials. By directly comparing Go and Stop trial RT distributions, SSRT should correspond to the earliest time point that Go and Stop trial RT distributions diverge and Stop trial RTs begin to significantly slow down.

This method was implemented in the following four steps: *First*, we drew *n* (where *n* is the number of stop trials) random samples (without replacement) from the approximately 2*n* Go trials in a session, and subtracted from these *n* sampled Go trial RTs the SSDs associated with Stop trials. This procedure created a new RT distribution such that Go trial RTs were re-aligned to would-be stop signals in order to compare with the Stop trial RT distribution also aligned to the onset of Stop signal (Figure 4A). *Second*, this sampling procedure was repeated 10,000 times to construct a conservative 99.9% (0.05–99.95%) confidence interval (CI) of the cumulative re-aligned Go trial RT distribution (Figure 4A). *Third*, we determined the earliest time point in the sorted Stop trial cumulative RT distribution at which RTs began to significantly slow down relative to the 99.9% CI (Figures 4A,B). To guard against false positive discovery from noisy Stop trial RT distributions, significant slowing of the Stop trial cumulative RT distribution should be present in at least ~0.15*n* consecutive Stop trials. This identified time point provided a WT cutoff that optimally separated failure-to-stop errors (which were statistically indistinguishable from Go trial RTs) from failure-to-wait errors (Figure 4A). This WT cutoff also determined the proportion of failure-to-stop errors, *p*(failure-to-stop), among all Stop trials in a session (Figure 4B). *Fourth*, the WT cutoff identified in the last step represented a conservative upper bound of the SSRT estimate, which was determined not only by the true SSRT but also affected by the number of Stop trials (*n*) and the chosen confidence interval (99.9%). To provide an unbiased estimate of the SSRT, we took the mean of the re-aligned cumulative Go trial RT distributions as the best estimate of re-aligned cumulative Go trial RT distribution, and determined the time point in this distribution that corresponded to the probability p(failure-to-stop) (Figure 4C). This time point was defined as the SSRT estimate because, according to the independent race model, the go process in *p*(*failure-to-stop*)^*^n trials would complete faster than SSRT and therefore escape inhibition. This procedure is conceptually equivalent to the commonly used integration method of SSRT estimation, where the time point in the Go trial RT distribution (relative to go signal onset) corresponding to *p*(failure-to-stop) is assumed to equal (SSD + SSRT). Thus, our method extended the integration method of SSRT estimation to RT distributions aligned to both actual and would-be stop signal onset. We therefore refer to this new method as the “modified integration method.”

This new method requires a few assumptions: *First*, Go trial RTs are generated by a stochastic go process that randomly draws from a static probability distribution represented by the observed Go trial RTs. It does not assume the shape of the RT distribution. *Second*, Stop trial RTs are generated by the same stochastic go process in competition with a stop process that begins to influence RT at the latency SSRT after stop signal onset. This assumption is validated by our empirical observation that the cumulative Stop trial RT distribution and the re-aligned Go trial RT distribution completely overlapped up until 130–150 ms after stop signal onset (Figures 4A,D). Note that, to ensure this assumption is met in practice, Go and Stop trials should represent independent and comparable sets of trials in the SST such that the stochastic go process is sampled equally in the two trial sets. *Third*, this method assumes that SSRT is independent of SSD so that data from all SSDs can be pooled together. This is not a stringent assumption because this method can be used to estimate SSRT for each SSD separately, but the fewer number of trials per SSD will dilute its power and make the estimate noisier. *Fourth*, this method does not assume that the Stop signal will necessarily slow down RTs. The method is in fact capable of detecting both significant RT speeding and slowing, but empirically we only observed significant RT slowing following the stop signal. This method also does not assume how the Stop signal will affect RT distributions *after* SSRT, it only works to detect the initial divergence between Go and Stop RT distributions. As a result, two animals can have the same SSRT estimate but one shows complete inhibition of behavioral responses after SSRT while the other shows a significant proportion of failure-to-wait errors.

### Median and integration methods of SSRT estimation

Both the median and integration methods require a binary classification of stop trials based on whether or not stopping was successful. This binary classification is needed to calculate the proportion of *failure-to-stop* errors, *p*(failure-to-stop), per SSD. The median method estimates SSRT by first fitting the inhibition function, SSD vs. *p*(failure-to-stop), commonly with logistic regression, to determine the SSD_50_ associated with *p*(failure-to-stop) = 0.5. SSRT is then estimated as the difference between the median Go trial RT (RT_50_) and SSD_50_. For sessions in which *p*(failure-to-stop) = 0.5 fell outside of the range of observed p(failure-to-stop), we did not provide a SSRT estimate to avoid errors in extrapolation. The integration method, on the other hand, estimates SSRT first for each SSD and then averages all estimates to produce the final SSRT estimate. For a particular SSD, e.g. SSD_100 ms_, the integration method finds *p*(failure-to-stop)_100 ms_th percentile in the Go trial RT distribution, RT_100 ms_, and estimates SSRT as RT_100 ms_ − SSD_100 ms_.

Critical to both methods is how stop trials are classified into binary outcomes, which is equivalent to setting a WT cutoff in Stop trials. Conventionally, successful stopping in rats is indexed by whether or not they obtained reward in each Stop trial, which is equivalent to setting the WT cutoff as the entire duration of the required hold period (500 ms). We explored varying the WT cutoff at different points of the hold period, as well as an ideal WT cutoff that perfectly distinguishes failure-to-stop errors from failure-to-wait errors in the RT simulation benchmark test (Figure 5). We also explored, with observed RTs in the SST (Figure 6), setting the WT cutoff as the conservative upper bound of the SSRT estimate determined in the third step of our method.

### RT simulation benchmark test

**Figure 2:**
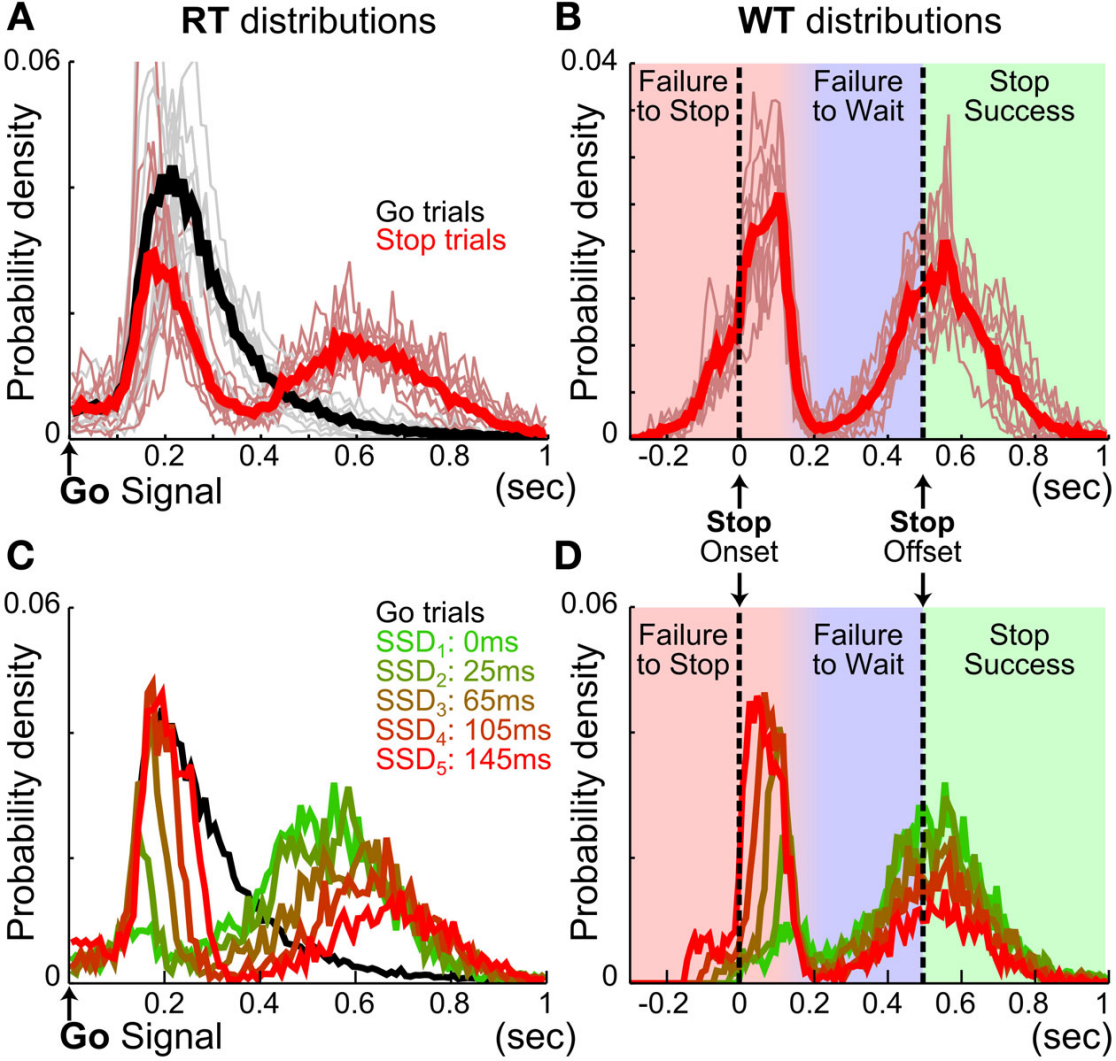
**Stop trial RTs are bimodally distributed. (A)** Mean RT distributions in Go (black) and Stop (red) trials. Thin lines represent the mean RT distribution for individual animals (*n* = 10). Stop trial RTs were bimodally distributed, with the fast RT peak largely overlapping with Go trial RTs. **(B)** Mean WT distributions in Stop trials. Thin lines represent the mean WT distribution for individual animals (*n* = 10). Vertical black dashed lines indicate onset and offset of the stop signal and correspond to the duration of the hold period. WTs were bimodally distributed, with the fast WT peak abruptly truncated within 150–200 ms after Stop signal onset. The color shadings indicate three types of Stop trials: failure-to-stop error (red), failure-to-wait error (blue) and stop success with reward (green). **(C)** Mean RT distributions in 95 sessions with the same SSDs for Go (black) and Stop (colored) trials. Stop trials were plotted separately for each SSD. This example illustrates that the relative proportion of the two RT peaks is a function of SSD. **(D)** WT distributions from the same Stop trials in **(C)** show that the fast WT peak is truncated at the same time point after stop signal onset irrespective of SSD.

To directly compare our new method of SSRT estimation with the two commonly used methods—median and integration methods, we simulated RT distributions in the SST using the independent race model with the SSRT fixed at 150 ms (Figure 5A). For each of the 100 simulation runs, we simulated 300 Go trials and 150 Stop trials. These 100 runs were blocked in 10 blocks of 10 model iterations each for statistical comparison. The go process was simulated by randomly drawing from a distribution equivalent to the mean of Go trial RT distributions across all sessions (from Figure 2A). The stop process was simulated by randomly drawing from one of 5 SSDs (0, 65, 105, 145, 185 ms) plus the duration of the preset SSRT (150 ms). Go trial RTs were determined only by the go process, while Stop trial RTs were determined by an independent race between the go and stop processes. If the stop process completed earlier than the go process, WT was then simulated by randomly drawing from a distribution corresponding to the second peak of the bimodal Stop trial WT distribution in Figure 2B. The WT distribution in Figure 2B was truncated at the trough between the two RT peaks (225 ms), and the area under the second peak was normalized to one. This second peak of the bimodal WT distribution corresponds to Stop trials that rats had successfully cancelled the planned go response, and may or may not have waited for the entire hold period to receive reward. Therefore, the ideal WT cutoff that perfectly separated failure-to-stop and failure-to-wait errors in this simulation was 225 ms. This simulation provided a close approximation of the empirically observed RT distributions in both Go and Stop trials, as well as the proportion of the two types of Stop trial errors. We used the simulated RTs to compare the accuracy and precision of SSRT estimation by all three methods against the preset SSRT (150 ms).

### Analysis of proactive inhibition

Proactive response adjustments were identified by comparing RTs on two Go trials (G_1_, G_2_) before and after a Go trial (G_1_-G-G_2_), a failure-to-stop trial (G_1_-F-G_2_), a failure-to-wait trial (G_1_-W-G_2_), or a successful stop trial (G_1_-S-G_2_). Only sessions with at least five trials for each of these four types of trial sequences were included in analyses (*n* = 184/257 sessions). The RT difference between G_2_ and G_1_ was reported as percent change in RT to quantify response adjustments (Beuk et al., 2014). In addition, G_1_ and G_2_ RTs were normalized to the median Go trial RT in order to examine absolute RT changes. Statistical comparisons were performed only between G_1_-G_2_ trials (Figure 7B). Intermediate (G, F, W, or S) trials were shown only for visualization and were not included in statistical analyses.

To investigate whether the frequency of Stop trials in recent trial history affects subsequent go response speed as well as stop success probability (Emeric et al., 2007; Ide et al., 2013), we identified sequences of seven trials that ended with a Go trial or a Stop trial (Figure 8). We classified trial sequences into different categories based on the number of stop trials in trials 1–6. For trial sequences ending with a Go trial, we normalized RTs in trial 7 associated with each trial sequence category by the session-wide median Go RT. A significant deviation of the median-normalized RT from 1 indicates a systematic fluctuation of RT based on recent trial history. For trial sequences ending with a Stop trial, we calculated the probability for each stop trial outcome—failure-to-stop, failure-to-wait and successful stop—in trial 7 associated with each trial sequence category. We determined whether the probability of stop success in each trial sequence category deviated from the session-wide stop performance after taking into account the contribution of SSDs. This was achieved by first determining the probability of each stop trial outcome associated with each SSD for the entire session, and then estimating predicted stop trial outcome probabilities in each trial sequence category based on the observed distribution of SSDs. The difference between the observed and predicted stop trial outcomes [Δ*p*(Stop)] represents the influence of recent Stop trial frequency on subsequent stop performance beyond what would be expected given the observed distribution of SSDs for that trial sequence category. Both median-normalized RTs and Δ*p*(Stop) were averaged across sessions within an animal to obtain estimates for each animal at each trial sequence category. Pearson correlation between normalized RTs and Δ*p*(Stop) was used to infer the coupling between these measures of proactive response adjustments.

Because some sequences of trials, especially those with very many (5–6) Stop trials, are uncommon, we only analyzed trial sequence categories with at least 5 sequences in a session. Furthermore, a trial sequence category in a session must have at least 5 sequences ending with a Go trial and 5 sequences ending with a Stop trial. To ensure a robust estimate, we further limited our analysis to trial sequence categories that were observed in at least 10% of sessions to minimize noise associated with very infrequent trial sequences containing many stop trials in a row. For this analysis, converted stop trials in trials 1–6 were considered as Go trials because the stop signal was not presented (i.e., for the animal, these trials were effectively Go trials). However, converted stop trials in trial 7 were considered as failure-to-stop errors to accurately estimate the entire range of stop trial outcomes. In addition, we did not constrain the order of trials in each sequence, only the number and types of trials. For instance, a trial sequence of Go-Stop-Go-Stop-Go-Stop-Go and Go-Go-Go-Stop-Stop-Stop-Go would both be similarly classified as having three stop trials preceding a Go trial.

### Analysis of trial sequence randomization

To better understand whether the pseudorandom trial sequences used in our experiment contained any embedded trial history dependency that might be exploited by rats, we compared the observed trial sequences with ideal pseudorandom trial sequences generated using MATLAB. Specifically, the pseudorandom trial sequences in our experiment were generated using the Med-PC function RANDD, which randomly draws from a pre-assigned 12-trial sequence containing 8 Go trials and 4 Stop trials without replacement. Simulated pseudorandom trial sequences were similarly generated using the MATLAB function randperm. Trial history dependency was analyzed using the autocorrelation coefficient (with MATLAB function xcorr) and by assigning Go trials as 1 and Stop trials as 0 in both observed and simulated trial sequences.

## Results

To develop a rodent-appropriate version of the SST, we incorporated two key elements in the primate SST that have not been consistently incorporated in other rodent SSTs: stopping the preparation of an action instead of stopping an ongoing movement (Eagle and Robbins, 2003a,b; Bari et al., 2011; Beuk et al., 2014), and the inclusion of multiple SSDs within a session (Eagle and Robbins, 2003a,b; Leventhal et al., 2012; Schmidt et al., 2013). Specifically, each trial was initiated by rats inserting their nose into a fixation port and waiting for an auditory go signal (Figure 1). On Go trials (67%), the go signal was presented alone and rats were rewarded in an adjacent port if the reaction time (RT), i.e., the latency between go signal onset and fixation port exit, was within 500 ms. On Stop trials (33%), the go signal was followed by a visual stop signal after a stop signal delay (SSD) randomly chosen from 5 possible latencies. In these trials, rats were rewarded only if the WT, i.e., the latency between stop signal onset and fixation port exit, was longer than 500 ms. Exiting the fixation port before the end of the hold period (WT < 500 ms) resulted in forfeit of reward. Successful performance in stop trials required rats to cancel the planned go response and maintain fixation for the entire hold period.

We first examined reactive inhibitory control in rats. Ten Long Evans rats were trained in the SST and were able to complete on average 354.08 ± 4.18 Go and 177.01 ± 2.10 Stop trials within a 90-min daily session (mean ± s.e.m. *n* = 257 sessions). Rats modulated fixation port exit RT based on trial type, making slower responses on Stop than Go trials [Go: 287.27 ± 18.27 ms, Stop: 431.04 ± 24.13 ms, *t*_(9)_ = −17.04, *p* = 4.0 × 10^−58^]. Closer examination of RT distributions shows that, unlike Go trial RTs, Stop trial RTs were bimodally distributed (Figure 2). The first and fast mode of Stop trial RTs closely overlapped with the Go trial RT distribution (Figures 2A,C), occurred well before the end of hold period, and were therefore considered as errors and not rewarded. This fast mode of Stop trial RT was abruptly truncated about 100–200 ms after the onset of the stop signal, which reflects a pause in behavioral response (Figures 2B,D). This was followed by the second and slower mode of Stop trial responses, with WTs centered on the end of the hold period (Figures 2B,D). A significant proportion of this second, slower mode of Stop trial responses occurred before the end of the 500 ms hold period and therefore were considered as errors and not rewarded. The consistent pattern of bimodal stop trial RT distributions across animals shows that two types of Stop trial errors are categorically different.

We hypothesized that the first type of Stop trial error corresponded to trials in which animals failed to stop the planned go response, and therefore the RTs were highly similar to Go trial RTs. We refer to this type of error as failure-to-stop (FS). We further hypothesized that the second type of error arose after animals had successfully stopped the initial go response, but failed to wait for the entire duration of the 500 ms hold period. We refer to this type of error as failure-to-wait (FW). According to this hypothesis, failure-to-stop errors in this task are analogous to non-cancelled trials in human and primate SST studies, while failure-to-wait errors would represent successful stopping but a failure of post-stopping related processing, suggesting the two error types may rely on separate neural mechanisms.

**Figure 3:**
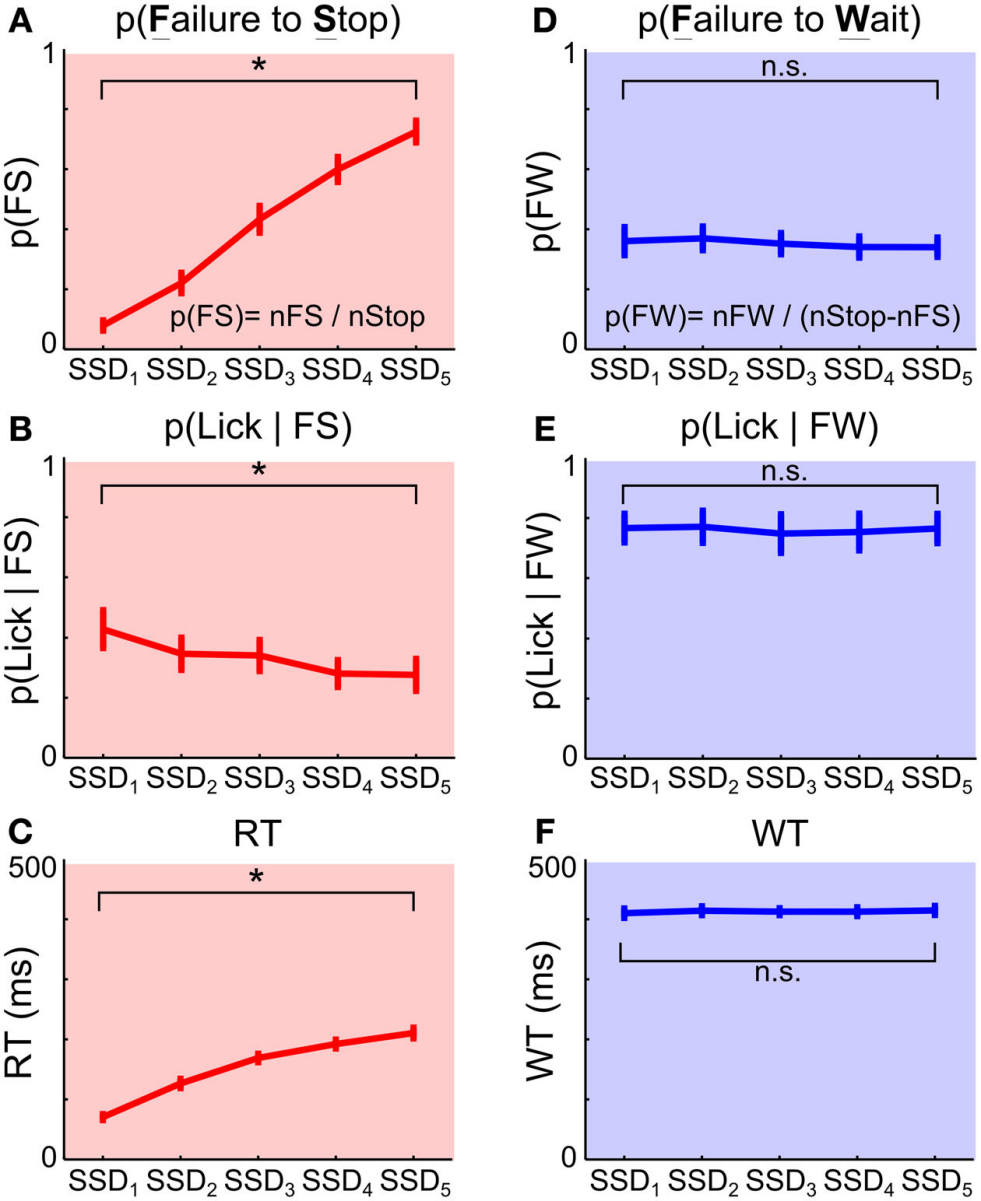
**Failure-to-stop and failure-to-wait represent independent errors. (A–C)** Failure-to-stop (FS) errors. **(A)** Proportion of FS trials among all Stop trials increased as a function of SSD, reflecting that stopping is harder when SSD is longer. **(B)** Proportion of FS trials in which animals attempted to lick for reward at the sipper tube. **(C)** RT relative to Go signal onset in FS trials increased as a function of SSD. **(D–F)** Failure-to-wait (FW) errors. **(D)** Proportion of FW trials among the subset of Stop trials in which the fast Go response was cancelled as a function of SSD. Unlike FS errors, FW errors were unaffected by SSDs. **(E)** Proportion of FW trials in which animals attempted to lick for reward at the sipper tube. Unlike FS errors, FW errors were associated with a high percentage of attempted reward collection and unaffected by SSDs. **(F)** WT relative to Stop signal onset in FW trials was not affected by SSDs. Error bars represent s.e.m (*n* = 10 rats). See Methods for the definition of FS and FW errors. Asterisks denote main effect of SSD (repeated measures ANOVA).

To test if the two types of Stop trial errors were independent of each other, we investigated whether the proportion and speed of each type of error changed as a function of SSD, and whether animals attempted to collect reward following each type of error. We found that longer SSDs were associated with higher percentages of failure-to-stop error [overall mean *p*(failure-to-stop) = 0.41 ± 0.03, main effect of SSD: *F*_(4, 36)_ = 175.14, *p* = 5.0 × 10^−24^, repeated measures ANOVA] (Figure 3A, see also Figures 2C,D). Furthermore, rats only attempted to collect reward in 33.44 ± 4.76% of these trials and were less likely to try to collect reward at longer SSDs [main effect of SSD: *F*_(4, 36)_ = 5.98, *p* = 0.0009, Figure 3B]. The mean RT in failure-to-stop errors increased with longer SSDs [overall mean = 153.77 ± 4.67 ms, main effect of SSD: *F*_(4, 36)_ = 102.27, *p* < 10^−20^] but were faster than the mean RT in Go trials [Go vs. failure-to-stop: 287.27 ± 18.27 vs. 153.77 ± 4.67 ms, *t*_(9)_ = 7.20, *p* = 9.1 × 10^−22^, Figure 3C]. These features are consistent with key properties of stop failure error predicted by the independent race model between the go and stop process (Logan and Cowan, 1984; Logan et al., 1984).

In contrast, the proportion of trials in which animals successfully stopped and subsequently failed to wait (failure-to-wait errors) was little affected by SSDs [Overall mean *p*(failure-to-wait) = 0.35 ± 0.04, no main effect of SSD, *F*_(4, 36)_ = 1.19, *p* = 0.33] (Figure 3D, see also Figures 2C,D). Rats attempted to collect reward in 76.00 ± 5.33% of these trials and attempted to collect reward with equal likelihood on all SSDs [no main effect of SSD, *F*_(4, 36)_ = 0.38, *p* = 0.82, Figure 3E]. Fixation port exit in these trials occurred close to the end of the 500 ms hold period and was not affected by SSD [mean *WT* = 412.85 ± 6.04 ms, no main effect of SSD, *F*_(4, 36)_ = 0.3, *p* = 0.88, Figure 3F]. Therefore, while rats similarly forfeited reward in these trials by responding during the 500 ms hold period, this mode of failure is distinct from the failure-to-stop error. In contrast to failure-to-stop errors, failure-to-wait errors are more similar to successful stop trials because of their long RTs near the end of the hold period and high percentage of reward collection attempts after fixation port exit. These properties support the idea that this type of error likely reflects failure-to-wait during the hold period, even though rats are able to successfully inhibit their planned go response.

**Figure 4:**
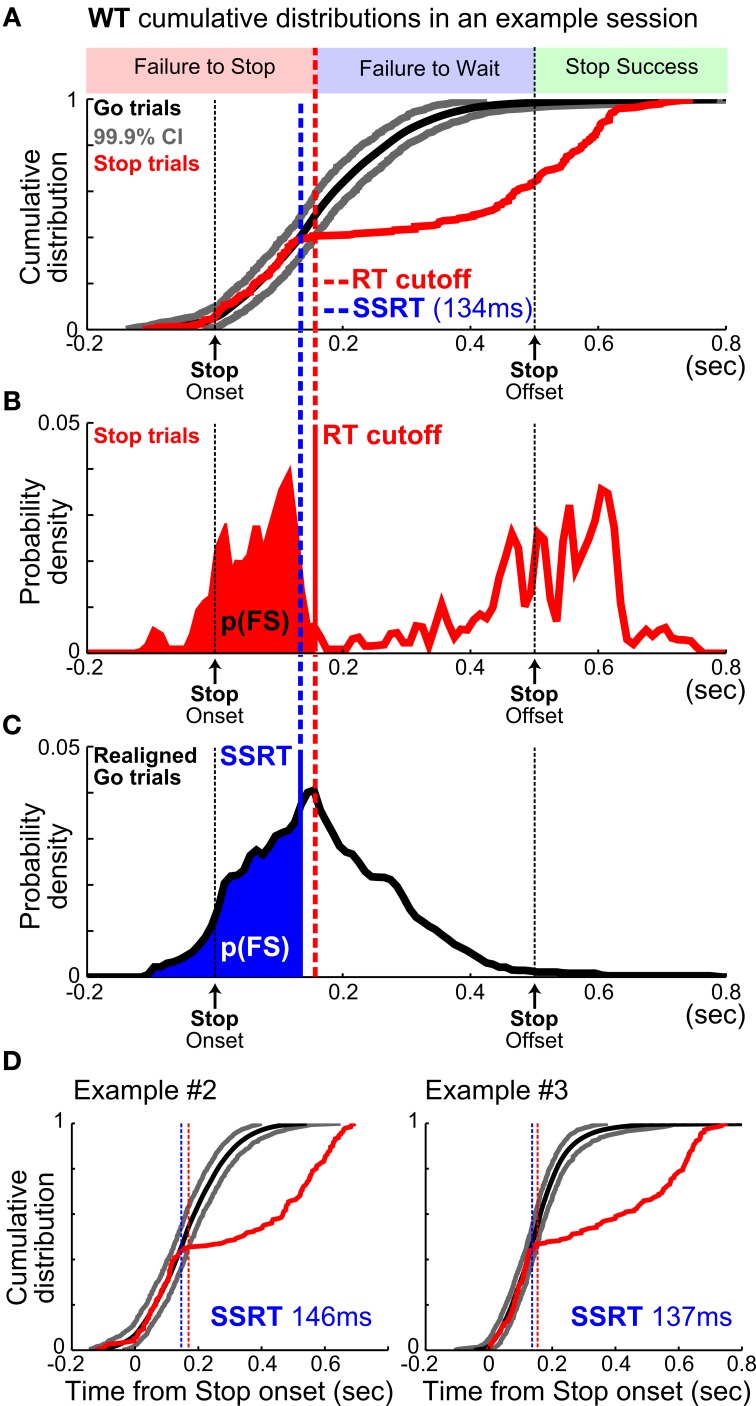
**A new method to estimate SSRT independent of failure-to-wait errors. (A)** Illustration of the new modified integration method by plotting the WT cumulative distributions from one example session. WT distributions relative to stop signal onset from Stop trials (red) and re-aligned Go trials (mean, black; 99.9% CI, gray). The intersection of Stop trial WTs (red) and the upper 99.9% CI bound (gray) defined an optimal WT cutoff (red vertical dashed line) that best separated FS and FW errors. **(B)** The WT cutoff determined the proportion of FS errors, p(FS), indicated by the red area under the WT distribution from Stop trials. **(C)** SSRT estimate (blue vertical dashed line) was defined as the time point in the WT distribution from re-aligned Go trials that corresponded to p(FS) under the curve. This is conceptually similar to the integration method of SSRT estimation, but aligned to stop signal onset. **(D)** SSRT estimation from two other example sessions. Note that WTs from re-aligned Go trials and Stop trials overlapped completely up until SSRT.

The prevalence of failure-to-wait errors poses a unique challenge of adopting the SST in rodents because SSRT should be estimated based only on failure-to-stop errors while excluding failure-to-wait errors, even though both types of error led to forfeiture of reward. However, these two types of errors have been traditionally conflated in rat studies because whether rats successfully obtained reward is typically used as a proxy for whether the planned go response was canceled. To disambiguate these two types of error, we developed a new analytic method that estimates SSRT independent of failure-to-wait errors. Because SSRT represents the time it takes to process the Stop signal and to cancel the planned go response, RTs in Go and Stop trials should be statistically indistinguishable up to the point of SSRT: RTs faster than this time point should similarly result from the execution of the go response alone (Logan et al., 1984) (Figure 4). Therefore, by directly comparing Go and Stop trial RT distributions, SSRT should correspond to the earliest time point that Go and Stop trial RT distributions diverge and Stop trial RTs begin to significantly slow down. We successfully implemented this method by extending the latency-matching procedure to generate Go trial RT distributions aligned to would-be stop signals, comparing the cumulative Go and Stop trial RT distributions, establishing statistical significance using a bootstrapped 99.9% confidence interval, and finally extending the integration method of SSRT estimation to RT distributions aligned to the onset of the stop signal (Figure 4). Importantly, this method does not require assumptions about the shape of RT distributions or how the Go and Stop process interact. Because of the conceptual similarity with the integration method, we refer to this new SSRT estimation method as the “modified integration method.”

**Figure 5:**
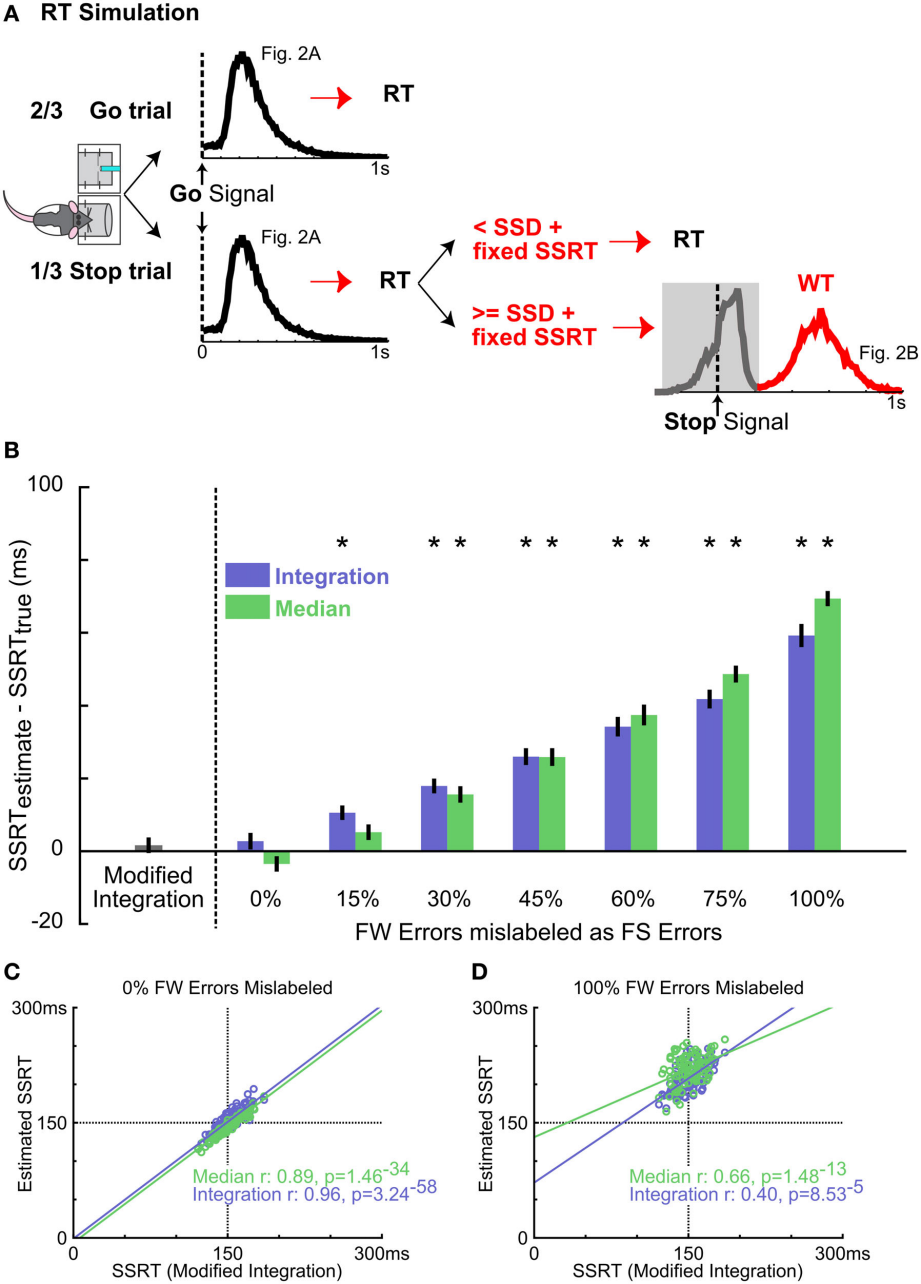
**Validating the new SSRT estimation method using simulated data. (A)** Schematic of the RT simulation. Each of the 100 simulation runs consisted of 300 Go trials and 150 Stop trials. RTs in Go trials were randomly drawn from the mean RT distribution from Figure 2A. Stop trials were modeled as an independent race between a go process (as in Go trials) and a stop process consisting of the SSD randomly drawn from 5 SSDs and a fixed SSRT at 150 ms. If the go process finished faster, RT was determined by the go process. If the stop process finished faster, the fast go response was inhibited and the WT was randomly drawn from the slow peak of the mean WT distribution from Figure 2B (red). This simulation produced RT and WT distributions similar to the empirical observation. **(B)** Summary of SSRT estimates (mean ± s.e.m) with varying amounts of FW errors mislabeled as FS errors for integration (blue) and median (green) methods, compared with SSRT estimated from the modified integration method. Conflation of FW errors as FS errors systematically overestimates SSRT using median and integration methods. Asterisks denote SSRT estimates significantly greater than the true SSRT (150 ms) by independent *t*-test Bonferroni corrected for multiple comparisons (*n* = 10.10-iteration blocks). **(C)** Scatter plot of estimated SSRTs over all model iterations between the modified integration method vs. the conventional median (green) and integration (blue) methods. For median and integration methods, the ideal WT cutoff (see Methods) was used to classify failed and successful stop trials. *n* = 100 iterations. **(D)** Scatter plot of SSRT estimates using the 500 ms WT cutoff, which mislabels all FW errors as FS errors. Convention as in **(C)** *n* = 100 iterations.

To validate our new modified integration method of estimating SSRT, we simulated RT distributions in the SST with a fixed SSRT of 150 ms (Figure 5A, schematic of simulation). Using the simulated RTs, we then compared SSRT estimation between the modified integration method and two commonly used methods—median and integration methods. RT distributions in the SST were simulated using the independent race model between the go process (randomly drawn from the empirically observed go RT distribution) and the stop process (randomly drawn from one of five SSDs plus the duration of SSRT). If the stop process completed earlier than the go process, WT was then determined by the empirically observed WT distribution in trials that rats had successfully cancelled the go response (corresponding to the second peak of the bimodal Stop trial WT distribution). Therefore, the simulation matches the empirically observed RT distributions, including the proportion of the two types of Stop trial errors.

Our simulation shows that the median and integration methods produced SSRT estimates that were, respectively, 69.60 ± 2.98 and 59.22 ± 3.02 ms (mean ± s.e.m.) slower than the true SSRT when success in Stop trials was defined as successfully waiting for the entire 500 ms hold period (i.e., 100% failure-to-wait errors mislabeled), as has been used in previous rodent SSTs (Figure 5B) (Eagle and Robbins, 2003b; Bryden et al., 2012; Leventhal et al., 2012). While overall SSRT estimates were similar between the median and integration method [no main effect of method, *F*_(1, 126)_ = 0.52, *p* = 0.64, repeated measures ANOVA, Method × Percent failure-to-wait Mislabeled], they were systematically overestimated with increasing inclusion of failure-to-wait errors [main effect of Percent failure-to-wait Mislabeled: *F*_(6, 126)_ = 193.02, *p* < 10^−20^, Figure 5B]. Troublingly, the mislabeling of as few as 15–30% of failure-to-wait errors (corresponding to 4–8 out of 150 Stop trials simulated) significantly biased the SSRT estimate by 10–20 ms [Median method SSRT_Estimated_ − SSRT_Actual_ ± s.e.m.; Median_15% failure-to-wait_: 6.48 ± 2.52 ms, *t*_(9)_ = 2.56, *p* = 0.0304, n.s. vs. Bonferroni corrected α/*n* = 0.0036; Integration_15% failure-to-wait_: 10.58 ± 1.90 ms, *t*_(9)_ = 4.31, *p* = 0.0003; Median_30% failure-to-wait_: 15.98 ± 12.49 ms, *t*_(9)_ = 6.42, *p* = 0.0001; Integration_30% failure-to-wait_: 17.94 ± 1.93 ms, *t*_(9)_ = 9.29, *p* = 7.0 × 10^−36^; Figure 5B]. The median and integration methods provided accurate estimates only when given an ideal WT cutoff that perfectly distinguishes failure-to-stop errors from failure-to-wait errors in the simulation (Figure 5B), which converged with the estimates of the modified integration method [Median method Pearson's *r*: 0.8865, *p* = 1.5 × 10^−34^; Integration method Pearson's *r*: 0.9641, *p* = 3.2 × 10^−58^; Figure 5C]. Using a cutoff that included 100% failure-to-wait errors significantly degraded this relationship (Median method Pearson's *r*: 0.6548, *p* = 1.5 × 10^−16^; Integration method Pearson's *r*: 0.4021, *p* = 8.5 × 10^−5^; Figure 5D). The modified integration method provided an accurate estimate of the true SSRT without the need for an arbitrary WT cutoff (Figure 5B). This comparison shows that the conflation of the two types of stop errors leads to significant bias in SSRT estimation using the conventional methods, and validates our new modified integration method in providing an unbiased SSRT estimate independent of failure-to-wait errors.

**Figure 6:**
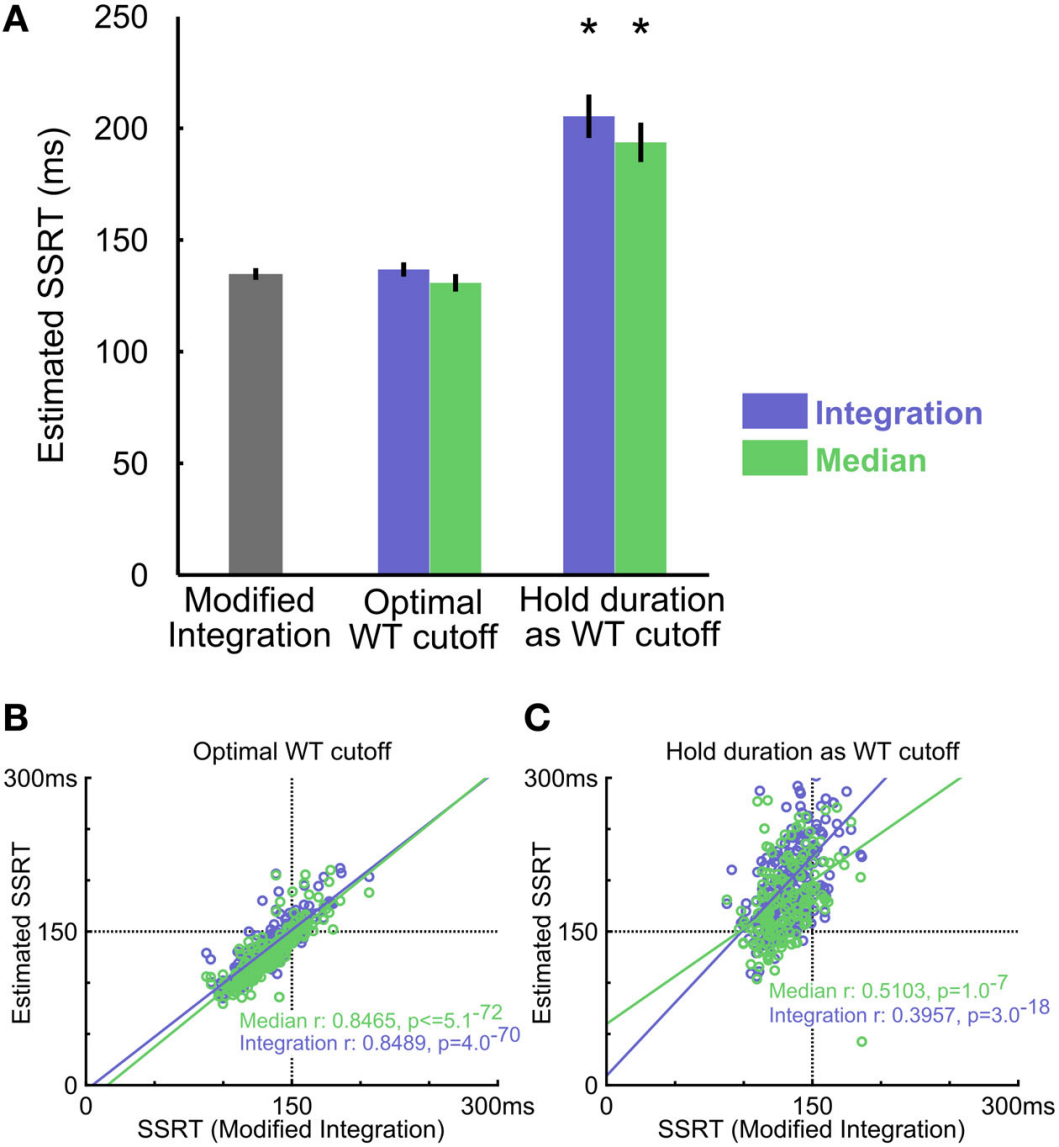
**Comparing three SSRT estimation methods in empirical data. (A)** Summary of SSRT estimates using different methods and WT cutoffs (mean ± s.e.m). The optimal WT cutoff refers to the conservative upper bound of the SSRT estimate (see Methods), while the hold period WT cutoff corresponds to defining success in stop trials as obtaining reward. Asterisks denote estimates significantly different from the modified integration method estimate corrected for multiple comparisons (*n* = 10 rats). **(B)** Scatter plot of estimated SSRTs over all behavioral sessions between the modified integration method vs. the conventional integration (blue) and median (green) methods, using the optimal WT cutoff. **(C)** Scatter plot of SSRT estimates using the entire hold period (500 ms) as cutoff, which mislabels all FW errors as FS errors. Convention as in **(B)** *n* = 257 sessions.

In empirical data from rats performing the SST, the modified integration method estimated SSRT at 134.77 ± 2.48 ms (mean ± s.e.m.), which was substantially faster than the estimates provided by median [193.72 ± 8.38 ms, *t*_(9)_ = −8.22, *p* = 1.8 × 10^−5^] and integration [205.40 ± 9.23 ms, *t*_(9)_ = −8.79, *p* = 1.0 × 10^−5^] methods using reward as the proxy for stop success (Figure 6A). As our simulation results illustrated, such differences in SSRT estimates likely resulted from the conflation of the two types of Stop trial errors. The modified integration method, however, can also provide a reliable WT cutoff that best separates the two types of stop errors such that median and integration methods produced similar SSRT estimates as the new modified integration method [Median method: 130.81 ± 3.72 ms, *t*_(9)_ = 1.86, *p* = 0.0953; Integration: 136.80 ± 3.03 ms, *t*_(9)_ = −1.19, *p* = 0.2632, Figure 6A]. The SSRTs estimated by median and integration methods using this optimal WT cutoff were highly correlated with the SSRT estimated by the modified integration method (Median method: Pearson's *r* = 0.8489, *p* = 5.1 × 10^−72^; Integration: Pearson's *r* = 0.8465, *p* = 4.0 × 10^−70^, Figure 6B), suggesting a convergence of SSRT estimation by all methods under ideal conditions. This correlation was significantly degraded when SSRT estimated by median and integration methods was based on reward as the proxy for stop success (Median method Pearson's *r* = 0.3957, *p* = 1.0 × 10^−7^; Integration method Pearson's *r* = 0.5103, *p* = 3.0 × 10^−18^, Figure 6C). These results suggest that SSRT estimates in rats have been significantly overestimated by existing methods when failure-to-wait and failure-to-stop errors are conflated.

**Figure 7:**
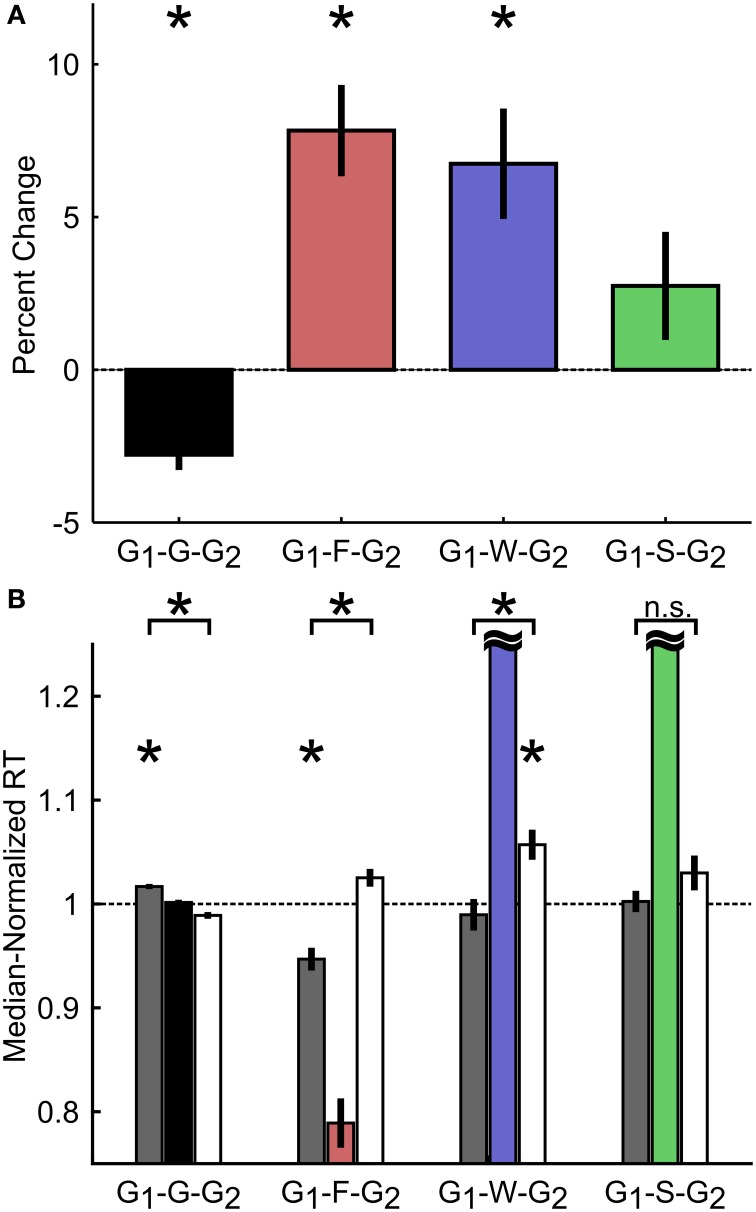
**Proactive adjustment of RT based on trial outcome. (A)** Percent change in RT between Go trials preceding (G_1_) and following (G_2_) different trial outcomes. G_1_-G-G_2_ refers to three consecutive go trials (F, failure-to-stop; W, failure-to-wait; S, stop success). **(B)** Median-normalized RT for the Go trials preceding (gray) and following (white) each trial outcome. Horizontal black dashed line indicates median RT. Intermediate trial data were included for visualization purposes only and not included in statistical analyses. Asterisks represent means significantly different than 1 or significant differences between groups, corrected for multiple comparisons. Error bars represent s.e.m. *n* = 10 rats.

Having demonstrated that rats exhibited reactive inhibitory control similar to primates, we further examined whether rats also employed proactive control strategies in the SST by adjusting the speed of their responses based on the outcome of previous trials (Emeric et al., 2007; Li et al., 2008b; Verbruggen and Logan, 2009; Ide and Li, 2011; Pouget et al., 2011; Bissett and Logan, 2012; Ide et al., 2013; Beuk et al., 2014). To this end, we first compared RTs in two Go trials (G_1_, G_2_) interleaved with either a Go trial (G), a failure-to-stop trial (F), a failure-to-wait trial (W), or a successful stop trial (S) as percent changes relative to G1 (Figure 7A) or as median-normalized RTs (Figure 7B). We observed that rats speed up following sequential Go trials (Median-normalized RT on G_1_-G trials: 1.02 ± 0.002; G-G_2_ trials: 0.99 ± 0.003; *t*_(9)_ = 5.86, *p* = 2.4 × 10^−4^ vs. Bonferroni corrected α/*n* = 0.0042). In addition, rats slowed down following both failure-to-stop [G_1_-F: 0.95 ± 0.01; F-G_2_: 1.03 ± 0.01; *t*_(9)_ = −5.52, *p* = 3.7 × 10^−4^ vs. corrected α/*n* = 0.0042] and failure-to-wait [G_1_-W: 0.99 ± 0.01; W-G_2_: 1.06 ± 0.01; *t*_(9)_ = −3.93, *p* = 0.0034 vs. corrected α/*n* = 0.0042] errors, but not successful stop trials [G_1_-S: 1.00 ± 0.01; S-G_2_: 1.03 ± 0.02; *t*_(9)_ = −1.64, *p* = 0.1365 vs. corrected α/*n* = 0.0042]. We further observed that, in Stop trials, only G_2_ trial RTs following failure-to-wait trials (W-G_2_) were significantly slower than the median RT [*t*_(9)_ = 4.15, *p* = 0.0025 vs. corrected α/*n* = 0.0042], while only G1 trial RTs before failure-to-stop trials (G1-S) were significantly faster than the median RT [*t*_(9)_ = −5.10, *p* = 0.0006 vs. corrected α/*n* = 0.0042]. These results suggest that, in proactive inhibitory control, not only do Stop trial errors induce modifications in subsequent response speed (G2), but also that the baseline response speed (G1) predicts whether subsequent stopping, but not waiting, would be successful.

**Figure 8:**
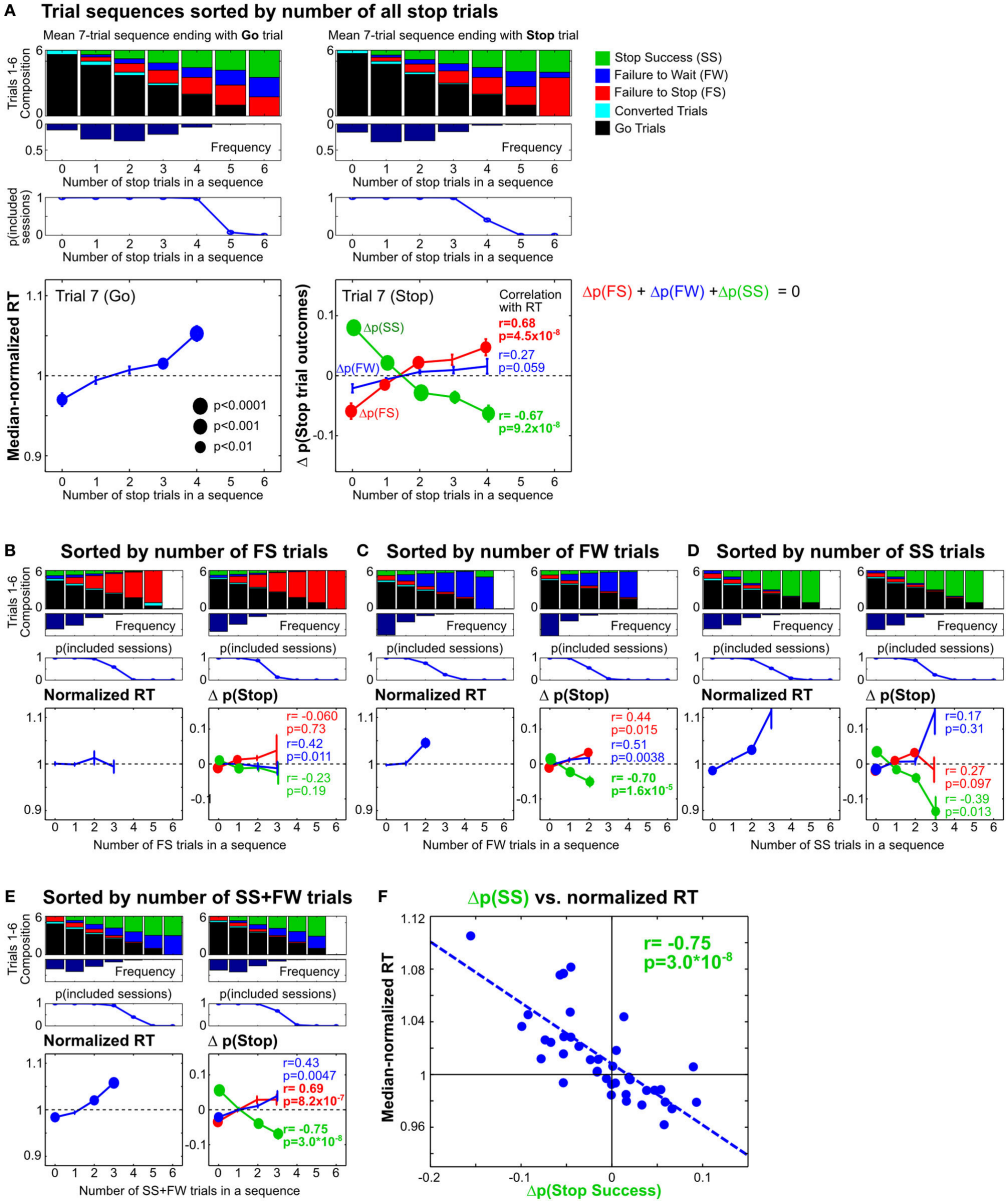
**Proactive adjustments of RT and stop performance by recent stop trials. (A)** Comparison of 7-trial sequences ending with a Go trial (left) or a Stop trial (right), sorted by the total number of all stop trials in trials 1–6. The top three rows show the average trial composition in trials 1–6 in each trial sequence category (top row), the respective relative frequencies (second row) and sessions containing at least 5 sequences in each trial sequence category (third row). The bottom row shows, for each trial sequence category, the median-normalized RTs (left) and the difference between the observed and predicted stop trial outcomes (right) in trial 7. The difference between the observed and predicted stop trial outcomes [Δ*p*(Stop)] represents the influence of recent Stop trial frequency on subsequent stop performance beyond what would be expected given the observed distribution of SSDs for that trial sequence category. *P*-values for *t*-test (*n* = 10) are represented by the size of circles. *R* and *p*-values for Pearson correlation between median-normalized RTs and Δ*p*(Stop) are indicated next to each type of Stop trial outcome. **(B–E)** The same analysis as in **(A)**, sorted by the number of failure-to-stop trials **(B)**, failure-to-wait trials **(C)**, successful stop trials **(D)**, or the number of successful stop trials and failure-to-wait trials combined **(E)**. **(F)** Correlation between median-normalized RTs and Δ*p*(Stop Success) based on trial sequence categories in **(E)**. These results show that proactive adjustments of RT and stop performance are highly correlated and jointly driven by recent failure-to-wait and successful stop trials.

Recent studies in humans and monkeys have also shown that the frequency of Stop trials in the recent trial history affect subsequent speed of go responses as well as the probability of stop success (Emeric et al., 2007; Ide et al., 2013). To further investigate whether similar effects are present in rats, we determined how Stop trial frequencies in any contiguous 6-trial sequence affected go and stop performance in the next trial, relative to the performance in the entire session. This was achieved by first identifying all 7-trial sequences that ended with a Go or a Stop trial (on trial 7), and categorizing trial sequences based on the number of Stop trials in trials 1–6 (Figure 8). We found that RTs on Go trials following no recent Stop trials were faster than median RT [*t*_(9)_ = −3.91, *p* = 0.0035 vs. corrected α/*n* = 0.01], while RTs following blocks of trials with 3/6 and 4/6 Stop trials were slower [*t*_(9)_ = 3.76, *p* = 0.0045 and *t*_(9)_ = 5.97, *p* = 2.1 × 10^−4^ vs. corrected α/*n* = 0.01] (Figure 8A, left). The pattern of proactive RT adjustment is consistent with other reports showing go RT speeding following infrequent recent Stop trials and go RT slowing following frequent recent Stop trials (Emeric et al., 2007; Ide et al., 2013) and also agrees with our observations in Figure 7. However, we found that frequent recent Stop trials were followed by worse stop performance compared to the session-wide performance level, and vice versa following infrequent recent Stop trials (Figure 8A, right). Furthermore, the extent of RT adjustment was significantly correlated with the extent of stop performance adjustment in the same trial categories (Figure 8A, right). In other words, frequent recent Stop trials resulted in slower response speed as well as worse stop performance in the following trial, and the extent of both adjustments were coupled. This observation was surprising because slower RTs are typically associated with better, not worse, stop performance, assuming that SSRT was not affected.

To further validate this surprising observation, and to delineate the respective contributions of three types of Stop trial outcomes, we sorted the same trial sequences by the number of failure-to-stop trials (Figure 8B), failure-to-wait trials (Figure 8C) and successful stop trials (Figure 8D). We found that trial sequence sorting based only on failure-to-stop trials did not result in significant RT modulations and only led to minimal stop performance adjustments that were not correlated with RTs, even in blocks with several failure-to-stop trials (Figure 8B). On the other hand, sorting based on both failure-to-wait and successful stop trials showed RT modulations and correlated stop performance adjustments (Figures 8C,D), patterns that were comparable to the findings in Figure 8A. These observations suggest that RT and stop performance adjustments were driven not by failure-to-stop trials but primarily by failure-to-wait and successful stop trials. Indeed, we observed the strongest correlation between RT and stop performance adjustments when trial sequences were sorted by the number of both failure-to-wait and successful stop trials (Figures 8E,F) (*r* = −0.75). Among the three possible Stop trial outcomes, the adjustment of stop success probability was consistently best correlated with RT adjustments. Together, the strong coupling between RT and stop performance adjustments suggests that both types of proactive adjustment were likely controlled by the same underlying mechanism. The commonality between failure-to-wait and stop success trials that were able to trigger these proactive adjustments is that both trial types involve successful stopping of the prepotent go response. Failure-to-stop trials, on the other hand, neither trigger proactive adjustments nor involve stopping of the go response.

**Figure 9:**
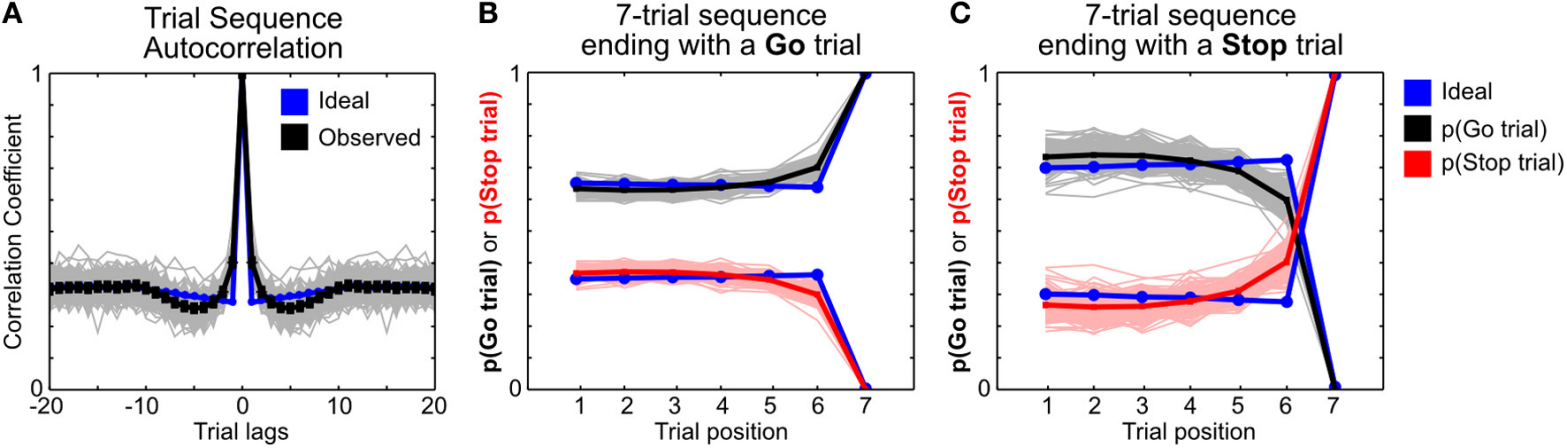
**Comparison of trial history dependencies in observed and ideal trial sequences. (A)** Autocorrelation of trial sequences in observed pseudorandom sequences generated by RANDD function from Med-PC IV (black) and in ideal pseudorandom sequences generated by MATLAB (blue). Trial sequences were converted into 1's (Go trials) and 0's (Stop trials). Each gray line represents the autocorrelation from one session, with mean ± s.e.m. of all sessions shown in black. **(B,C)** The frequencies of Go and Stop trials in 7-trial sequences ending with a Go trial **(B)** or a Stop trial **(C)**, with observed frequencies in black (Go) and red (Stop) and the corresponding ideal frequencies in blue. There is a small but significant non-random trial history dependency in the observed trial sequences, suggesting imperfect pseudorandomization by RANDD.

Finally, to ensure that the proactive adjustments we observed did not result from any embedded trial history dependency in our experiment that rats may be able to exploit, we compared the observed trial sequences with simulated ideal pseudorandom trial sequences. We found that the randomization function used in the current study (RANDD function from Med-PC IV) contained a small but significant non-random trial history dependency that deviated from ideal pseudorandom sequences (Figure 9). The experimental trial sequence introduced a small above-random correlation in adjacent trials (trial lag 1 in Figure 9A), such that a Go trial is more likely to be preceded and followed by a Go trial, and that a Stop trial is more likely to be preceded and followed by a Stop trial (Figure 9B). While we cannot rule out that rats may exploit this trial history dependency and such behavioral strategies may contribute to the observed proactive adjustments, we believe such contributions are minimal for the following reasons. If rats were able to detect and exploit the trial history dependency, they should expect to encounter Stop trials following frequent recent Stop trials and use this predictive information to slow down response speed as well as increase stop success probability. While the observed RT adjustment was consistent with this interpretation, the stop success probability did not increase as predicted but instead decreased. We therefore believe that the observed proactive adjustments occurred in spite of the non-random trial history dependencies.

## Discussion

In this study, we developed a novel, rodent appropriate SST and characterized both reactive and proactive control strategies in rats. Our rodent SST incorporated key elements commonly used in the primate SSTs, including multiple SSDs and requiring subjects to cancel a planned action instead of stopping an ongoing movement (Figure 1). For reactive inhibitory control, we showed that errors in Stop trials resulted from two independent sources: failure-to-stop the go response, or failure-to-wait after stopping was achieved (Figures 2, 3). The conflation of these two types of errors systematically overestimates SSRT by at least 50ms both in a simulated race model and in practice (Figures 5, 6). To address this issue, we developed and validated a novel modified integration method that provides an unbiased SSRT estimate independent of the ability to wait (Figures 4, 5, 6). For proactive inhibition, we found that rodents adjust both RTs and their ability to stop following failure-to-wait and successful Stop trials, but not after failure-to-stop trials (Figures 7, 8). Together, these results establish the rat as a valid model that displays proactive and reactive inhibitory control as observed in monkeys and humans.

Our study differs from previous attempts to translate the SST to rodents in several critical ways. While rats are required to cancel the initiation of an action in our task as in most primate and human studies, previous rodent studies from both Eagle et al. (Eagle and Robbins, 2003a) and Beuk et al. (2014) used a task in which rodents were required to inhibit an ongoing movement following a stop signal. It is not clear if these experimental designs invoke the same task demands as in human and primate versions of the SST. While this is not a concern for Berke et al. (Leventhal et al., 2012; Schmidt et al., 2013), which required rats to cancel a fixation port exit response similar to the task design used here, their paradigm, as well as those used by Eagle and colleagues, used only a single, constant SSD per session. The practice of using a constant SSD makes the stop signal highly predictable and likely encourages rats to employ a timing strategy to improve stopping. Such a concern is minimized in primate SSTs, which typically use multiple SSDs or a tracking procedure to dynamically adjust SSD across trials. Our SST design addresses both issues and successfully translates the SST to rodents.

In SST studies of humans and non-human primates, proactive and reactive control each refers to preparation to stop prior to signal onset and stimulus-driven processing at signal onset. In the context of understanding reactive inhibitory control in rodents, our results highlight the unique challenge in rodents to dissociate initial stopping from subsequent waiting in the SST. While the stop signal similarly instructs humans, monkeys and rats to withhold the prepotent go response for a short period of time (the hold period), rats are especially prone to committing errors during this period. Further, while failure-to-stop errors likely represent failures of reactive control (Figures 3A–C) and are influenced by proactive adjustments (Figure 7), failure-to-wait errors seem unaffected by SSD, RT, or proactive adjustments (Figures 3D–F, 7). This difference likely originates from two sources: First, rodents may be more impulsive than primates and less able to wait for the entire duration of the hold period after they have successfully stopped the planned go response (Robinson et al., 2009). Second, the nature of behavioral responses generated by primates and rats in behavioral tests are fundamentally different and these differences must be accounted for. For instance, unlike in primates, rats needed to rapidly exit the fixation port to approach, collect, and consume the reward after they had successfully stopped the planned go response and waited for the entire hold period. In contrast, reward in primate SSTs is not directly associated with specific task-relevant behavioral responses such as saccade or button press, but is instead generally delivered without an instrumental requirement (e.g., licking). Therefore, in rodents this reward-approaching response likely commands a stronger motivational drive than in primates, especially toward the end of the hold period when reward availability is imminent.

Our results support that failure-to-stop and failure-to-wait errors are generated independently. We show that failure-to-wait errors are associated with long WTs and a high frequency of reward collection attempts, but are not modulated by SSD (Figure 3), properties that are very similar to successful Stop trials. By contrast, failure-to-stop errors are significantly modulated by SSD and their associated RTs are faster than Go trial RTs (Figure 3), which recapitulate the key properties predicted by the independent race model (Logan and Cowan, 1984; Band et al., 2003). These findings are consistent with the interpretation that failure-to-stop errors result from failure of reactive inhibition to cancel the planned go response, while failure-to-wait errors represent premature attempts to collect reward after stopping was successful and are unrelated to factors affecting stopping. The presence of failure-to-wait errors likely represent a common feature of rodent SSTs that must be addressed when estimating SSRT (Eagle and Robbins, 2003a; Bryden et al., 2012; Leventhal et al., 2012; Schmidt et al., 2013). For example, RTs on failed Stop trials are similarly bimodally distributed in Figure 1 of Schmidt et al. (2013) and Figure 4B of Leventhal et al. (2012). The distinction between failure-to-stop and failure-to-wait errors is important because these two types of errors are under the control of separate behavioral variables and therefore likely arise from distinct underlying neural mechanisms. In addition, our rodent SST may also serve as a useful model to independently assess two forms of impulsive action in a within-task design (Eagle et al., 2008; Broos et al., 2012; Worbe et al., 2014).

It is important to note that the distinction between failure-to-stop vs. failure-to-wait errors does not map onto the distinction between proactive and reactive inhibitory control. Rather, distinguishing failure-to-stop from failure-to-wait errors is relevant primarily for correctly estimating SSRT, which embodies the reactive inhibitory control process. It is also important to note that, while our study identifies and establishes failure-to-wait errors to be distinct from failure-to-stop errors, it remains unclear whether failure-to-wait errors are also present in human and primate studies or whether failure-to-wait errors represent a rodent-specific phenomenon.

The conflation of these two types of errors in rodent studies not only significantly overestimates SSRT (Figures 5, 6), such conflation may lead to incorrect mechanistic interpretations because manipulations such as psychostimulant administration (Fillmore and Rush, 2002; Li et al., 2006b, 2008a, 2010; Groman et al., 2009; Liao et al., 2014) or prefrontal cortical lesion (Aron et al., 2003; Picton et al., 2007) may also affect impulsivity (Mendez et al., 2010; Mar et al., 2011) and therefore the ability to wait. In such cases, manipulations that affect the ability to wait will produce longer SSRT estimates when the two types of stop error are conflated, and likely would be misinterpreted as affecting the ability to stop instead (Farr et al., 2012). Even in cases in which such manipulations are not used, the mislabeling of as few as 15% failure-to-wait errors (corresponding to about 3% of all stop trials) as failure-to-stop errors significantly inflates the SSRT estimate. Given the rapidly growing demand for an appropriate rodent SST to link the computational power of the SST to techniques too costly or not yet available in human and monkey studies, special attention must be placed on ensuring rodent SSRT estimates are valid, reliable, and comparable to primate estimates.

The new method developed in this study represents an extension of the commonly used integration method, and provides an unbiased SSRT estimate independent of the ability to wait, without assuming the shape of RT distributions or how the go and stop process interact (Figures 4, 5). This modified integration method estimates SSRT by comparing RT distributions in Stop trials with appropriately resampled and realigned Go trials to would-be stop signals. The modified integration method provides a direct parallel with, and was in fact inspired by, neurophysiological data analysis where responses are aligned to the onset of distinct events. Therefore, the modified integration method can potentially provide a unified framework for comparing behavioral and neural responses between Go and Stop trials, a major analytic advance applicable to rodents and primates alike.

Using this new modified integration method of SSRT estimation, we found that rats show a very robust and fast stopping behavior. Our estimate of SSRT is ~50–60 ms faster than SSRT estimates using median or integration methods when the failure-to-stop and failure-to-wait errors are conflated, as has been the case in previous rodent studies (Eagle and Robbins, 2003a; Bryden et al., 2012). The use of a long RT cutoff to separate failure-to-stop from failure-to-wait errors by Leventhal et al. offered a sub-optimal solution and similarly produced substantially slower SSRT estimates than reported here (162.00 ± 12.65 vs. 134.77 ± 2.48 ms). This bias represents a significant percentage of the SSRT estimate and is of critical importance for existing and future rodent studies using the SST. More importantly, such a bias may also shift the temporal order and causal inference between neurophysiological responses and SSRT, such that a neural signal that was faster than conventional SSRT estimates in rodents may in fact occur after the true SSRT. Special attention must be paid to minimizing or eliminating the conflation of failure-to-stop and failure-to-wait errors.

Our analysis using RT simulations further shows that conventional methods of SSRT estimation can be salvaged by selecting an optimal WT cutoff that best separates failure-to-wait error from failure-to-stop error (Figure 5). While setting such a WT cutoff in empirical datasets can be subjective and arbitrary because substantial differences exist between subjects and between different training sessions within the same subject, our new modified integration method can also provide an unbiased WT cutoff so that SSRT estimates from all methods converge (Figures 5, 6).

In the context of understanding proactive inhibitory control in rodents, we demonstrate that like primates, rats employ a proactive slowing strategy based on recent trial history (Rieger and Gauggel, 1999; Emeric et al., 2007; Verbruggen and Logan, 2009; Nelson et al., 2010; Pouget et al., 2011). In 3-trial sequences, we observe that RTs are slower than the median RT following failure-to-wait errors (Figure 7B). In addition, we observe that RT on the previous trial predicts the outcome on the current trial: RT prior to a failure-to-stop trial was faster than the median RT, while RT prior to a successful Stop or failure-to-wait trial was not different from the median RT. These data suggest a complicated push-pull relationship in rats between the predisposition to respond quickly and the task-relevant requirement to stop. We extend those findings in the longer 7-trial sequence (Figure 8) and observe that RTs following frequent recent Stop trials are slower than the median RT, while RTs following infrequent recent Stop trials can be faster than the median RT. The presence of significant RT adjustment following stop success trials in Figure 8 but not in Figure 7 likely resulted from differences in trial sequence lengths and trial sequence compositions between the two analyses.

Much to our surprise, however, the same trial sequences leading to slower subsequent RTs are associated with worse stop performance relative to the session-wide stop performance level, even after controlling for the contribution of SSDs (Figure 8). Such an adjustment pattern cannot be explained by the non-random trial history dependencies that we uncovered (Figure 9). In the context of the independent race model, stop performance is completely determined by the go RT distribution, SSRT and SSDs. Therefore, our observation that slower RTs are associated with worse stop performance necessarily implies that SSRT slows down even more so than RT slowing. Together, these observations imply that, rather than being stationary stochastic processes, both the Go RT distribution and the SSRT are subject to trial-by-trial variation in their underlying parameters and are dynamically adjusted based on recent trial history. While such dynamic adjustments pose a serious challenge for accurately estimating SSRT, our analysis shows that a comparison with the session-wide performance level provides a viable way to identify systematic dynamic adjustments in stop performance based on local trial history.

Two observations are important for interpreting the surprising finding that slower RTs following frequent recent stop trials are associated with worse stop performance. First, the extent of stop performance adjustment is tightly coupled with the extent of RT adjustment, suggesting that both effects arise from a common cause (though, importantly, they may not share a common neural mechanism). Second, both RT and stop performance adjustments are specifically driven by failure-to-wait errors and successful stop trials but not failure-to-stop errors, with the former two sharing the common feature that the prepotent planned go response is successfully canceled. Such an observation also supports our conclusion that failure-to-wait errors are similar to successful stop trials and distinct from failure-to-stop errors with which they should not be conflated.

One possible hypothesis based on these observations is that the common driving force for both types of proactive adjustments is the conflict between the go response rule and the stop response rule in the context of relatively common Go trials and relatively infrequent Stop trials. In other words, following successful and repeated execution of stopping in recent past trials that takes place against the background of low Stop trial probabilities, rats are less certain about which of the two response rules to prepare for: the global and more probable go response or the recent but less probable stop response. As a result, rats are less efficient at executing both the go and the stop response. Such a conflict is not present, however, in failure-to-stop errors when rats failed to execute the stop response. Future studies are needed to reconcile the differences between the current finding with those in humans and non-human primates (Ide et al., 2013).

Together, the current study establishes the rat as a valid model that displays both proactive and reactive inhibitory control similar to monkeys and humans. This is especially important because SSRT estimates are elevated in individuals with deficient cognitive control, including adults (Bekker et al., 2005) and children (De Zeeuw et al., 2008) with Attention Deficit Hyperactivity Disorder, Obsessive-Compulsive Disorder (Lipszyc and Schachar, 2010), Parkinson's Disease (Mirabella et al., 2011), pathological gambling (Grant et al., 2011), Tourette syndrome (Goudriaan et al., 2005; Ray Li et al., 2006), schizophrenia (Hughes et al., 2012), drug abuse (Li et al., 2006b, 2010; Liao et al., 2014) and normal cognitive aging (Kramer et al., 1994; Coxon et al., 2012; Hu et al., 2013). The current study therefore represents an important first step in connecting the quantitative power of the SST paradigm for studying inhibitory control with the unique advantages of dissecting neural circuit mechanisms in rodent models to advance research with both basic science and clinical implications.

### Conflict of interest statement

The authors declare that the research was conducted in the absence of any commercial or financial relationships that could be construed as a potential conflict of interest.
